# Supplementary material for: Changes in Body Mass Index after Initiation of Antiretroviral Treatment: Differences by Class of Core Drug
Source: Viruses. 2022 Jul 29;14(8):1677. doi: 10.3390/v14081677 (PMC9415309; doi:10.3390/v14081677)
Supplement: Supplementary file 1 [file viruses-14-01677-s001.zip › viruses-1773934-supplementary.pdf]

Table S1. Differences in demographic and clinical characteristics between participants in the study sample and participants excluded due to missing baseline data.

**Variable**	**Excluded**	**Included**	
	n=1608 (62.08%)	*n=982 (37.92%)*	**p-value**
ART regimen category			0.406
Boosted PI[1]	618 (38.43%)	352 (35.85%)	
NNRTI[2]	578 (35.95%)	364 (37.07%)	
INSTI[3]	412 (25.62%)	266 (27.09%)	
Age at ART initiation (years)	35.9 (30.3, 44.0)	35.2 (29.7, 42.9)	0.010
Female sex	202 (12.56%)	80 (8.15%)	<0.001
Transmission mode			<0.001
MSM[4]	1026 (63.81%)	665 (67.72%)	
PWID[5]	170 (10.57%)	129 (13.14%)	
Heterosexual	292 (18.16%)	144 (14.66%)	
NA[6]	120 (7.46%)	44 (4.48%)	
Ethnic/racial group	10.00 (10.00, 10.00)	10.00 (10.00, 10.00)	<0.001
White	1492 (92.79%)	918 (93.48%)	
Black	79 (4.91%)	9 (0.92%)	
Asian	10 (0.62%)	3 (0.31%)	
Middle East/Arab	3 (0.19%)	0 (0.00%)	
NA[6]	24 (1.49%)	52 (5.30%)	
Year of ART initiation	2013 (2011, 2015)	2013 (2011, 2015)	0.176
AIDS before baseline	141 (8.77%)	42 (4.28%)	<0.001
Baseline[7] CD4 (cells/μl)	306 (180, 417)	325 (212, 428)	0.002
Baseline[7] HIV-RNA (log_{10} c/ml)	4.50 (3.86, 5.10)	4.50 (3.94, 4.99)	0.769

[1]Protease Inhibitors; [2]Non-Nucleoside Reverse Trascriptase Inhibitors; [3]Integrase Strand Inhibitors; [4]Men having sex with men; [5]People who inject drugs; [6]Non-Available; [7]At ART initiation, available for 1498/1608 (CD4) and 1525/1608 (HIV-RNA).

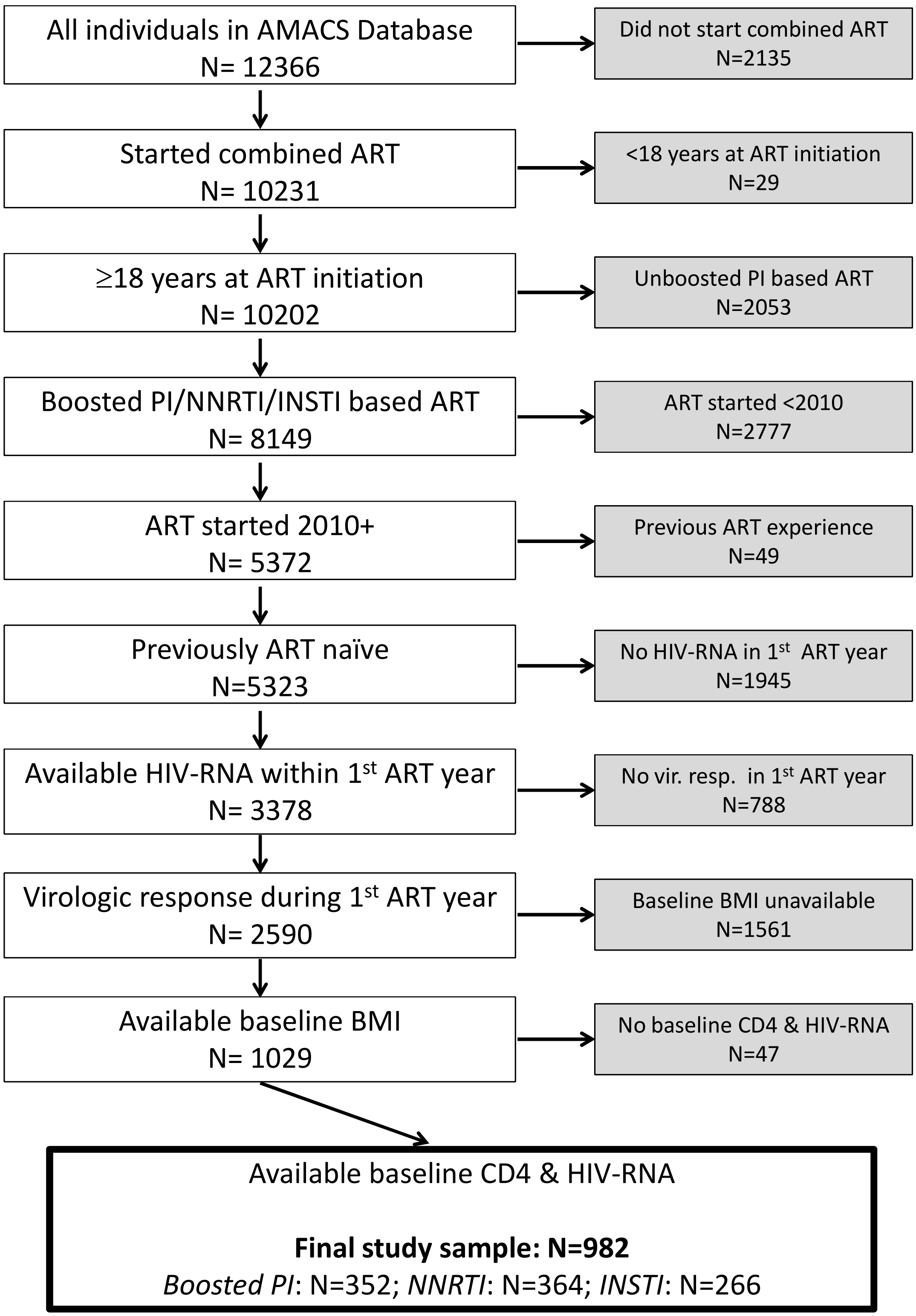

Figure S1. Flowchart for inclusion/exclusion criteria.
